# Recent Investigation into the Pathology of Tetanus and the Production of Immunity in Animals

**Published:** 1891-10-17

**Authors:** 


### SURGERY.

### RECENT INVESTIGATIONS INTO THE PATHOLOGY OF TETANUS AND THE PRODUCTION OF IMMUNITY IN ANIMALS.

The symptoms of tetanus so much resemble those of strychnine poisoning that for some time before a bacillus was discovered in connection with the disease, it was supposed they might be due to the absorption of some poison generated in the wound. We now know that this is so, and that the poison is a substance produced by the tetanus bacilli just as tuberculin is the product of the tubercle bacilli.

The history of the discovery of the tetanus bacillus is a very interesting one, as the discoverer was not investigating the pathology of that disease when he found the bacillus. He was experimenting on the effects of earth introduced under the skin of animals, and he found that earth from some places produced tetanus, whilst earth from other parts produced malignant œdema. In the animals in whom tetanus developed a bacillus having a characteristic mode of spore formation was found in the pus which formed at the seat of inoculation, and this pus when injected into other animals proved more virulent than the culture which originally produced the disease. The characteristic spore formation consists in the rounded enlargement of one end of the bacillus where the spore is formed, so as to give it the appearance of a drumstick. But the tetanus bacillus is not the only one of this shape known; there is another of similar form present in some processes of putrefaction, and therefore it, like most bacilli, is only known by its effects. Nicolaier could not separate this bacillus from the others with which it was mixed in the wounds and in cultures made from the wounds, so that he was unable to prove that it, apart from the other bacteria, was the cause of tetanus. But Kitasato accomplished this difficult task by a very ingenious method. The tetanus bacillus spores earlier than any other form with which it grows in the culture, and so it occurred to him that he might at an early period, when only the tetanus bacillus had developed spores, kill all the bacilli by a degree of heat which would not injure the tetanus spores, and then inoculate a new medium with them, and thus obtain a pure cultivation. This pure cultivation he found produced the disease when injected into animals, and so the identification of the tetanus bacillus was complete.

This pure cultivation has a characteristic form, and the gelatine in which it grows a peculiar fusty smell. As it develops it presents the appearance of a spruce-fir, the lower branches being longer and more distinctly marked than those nearer the surface.

But for some time the chain of evidence which proved the bacillus to be the cause of tetanus was incomplete. Cultivations injected into animals failed to produce the disease, and inoculations of pus from wounds of tetanus patients also failed. This seemed to disprove the relation of the bacillus to the disease: but after a time it was discovered that whenever it grew in the presence of oxygen, either on plate cultivations or in the pus of open wounds, it did not develop any power of producing the disease. When, however, it was grown in an atmosphere of hydrogen, or below the surface in the gelatine nutrient medium, it was very virulent. And yet inoculation with even pure cultures grown under anærobic conditions often failed to cause the disease. Recently, however, it has been shown that it is not the organism but the chemical poison produced in its growth that causes tetanus, and this poison, even in pure cultures, the bacilli take some time to produce.

Another link in the chain of evidence seemed also to be wanting. Although earth inserted under the skin of animals produced tetanus, yet no tetanus bacilli were ever found in it. Nevertheless it was shown that sterilisation of the earth prevented the inoculation producing the disease, and hence we are justified in concluding that although the bacilli are not present yet their spores are. And these spores are now found to have a much wider distribution than garden soil. Successful inoculations have now been made from the sweepings of a hay loft and the dust from horses' furniture, and the specific bacillus has been found in the dirt on a man's hand and imperfectly cleansed surgical instruments. M. Bossano inoculated animals with soil from forty-three different regions in various parts of the globe, and in twenty-seven animals tetanus developed. This shows how wide is the distribution of the bacillus in soil. There is little doubt that this is the poison used by some tribes on poisoned arrows. Where arrows have been charged by covering the point with soil from a mangrove swamp, tetanus has been reported to follow in all those injured by the poisoned weapons.

Remembering how often the tetanus bacillus is present in earth we cannot be too careful in speedily and thoroughly removing it from all recent wounds. This is especially so with regard to cultivated soil containing much organic matter, for such soil almost invariably contains the bacilli.

It is a curious circumstance that although the tetanus bacilli cannot grow as a virulent organism where there is free oxygen, yet, on the other hand, it never penetrates far from the wound, as is also the case with the bacillus in diphtheria. It seems to grow in the superficial layer of the wound, protected from oxygen by the leucocytes of the pus which absorb it, on the one hand, and unable to encounter the oxygen of the blood in the vessels in the deeper part of the wound on the other. It is never found in the internal organs, or blood of the animals in whom the disease is artificially produced, although present in the pus at the seat of inoculation. We have recently examined the blood from a case of tetanus, but failed to find any bacillus present. And yet inoculation with the blood and portions of internal organs will produce the disease, because, although no bacilli are present, yet they are saturated with the chemical poison produced by the growth of the bacilli in the wound. This chemical poison has been separate. A very virulent ptomaine and poisonous toxalbumen were obtained from the limb of a patient who died of tetanus, and it is probably the action of these poisons on the nervous system or muscles that gives rise to the symptoms of the disease.

We must regard, then, the wound of a patient suffering from tetanus as the manufactory of a deadly poison; to be most thoroughly cleansed and disinfected. But ought we not to do more than this? However we may purify the wound, we must remember the bacilli grow virulent, not so much in the pus as in the superficial layer of the tissue, and we cannot help thinking that the more reasonable procedure would be to excise the wound or amputate the limb. But, unfortunately, we know that this has seldom succeeded in the past. Probably so much poison has been absorbed that removal of the bacteria infected wound is useless. The prevalence of tetanus is known to have been lessened by antiseptic treatment of wounds. The tissues in a healthy aseptic wound are probably better able to contend with the organism if it has gained entrance, and to destroy it before it develops its poison.

And here we come to the question of immunity, a more hopeful one as regards the future prospects of treatment. Whatever credit we may give to the phagocytes in the wound for destruction of the bacilli it is on the chemical constitution of the blood and tissues we must at any rate to a large extent rely. Behring and Kitasato have shown that rabbits may be rendered artificially immune, by the injection of trichloride of iodine, and that the blood of animals thus rendered immune will, when injected into others, arrest the disease. That tetanus can be prevented and arrested by the injection of an inorganic salt is surely one of the most hopeful therapeutic discoveries of modern times, and this we owe to bacteriology.

